# The estimated number of potential PrEP users among gay-identifying men who have sex with men in Australia

**DOI:** 10.1371/journal.pone.0204138

**Published:** 2018-10-18

**Authors:** Iryna B. Zablotska, Richard Gray, Bill Whittaker, Martin Holt, Edwina Wright, Garrett Prestage, Darryl O’Donnell, Andrew E. Grulich

**Affiliations:** 1 Faculty of Medicine and Health, the University of Sydney, Sydney, New South Wales, Australia; 2 The Kirby Institute, UNSW Sydney, Sydney, New South Wales, Australia; 3 National Association of People with HIV, Newtown, New South Wales, Australia; 4 Centre for Social Research in Health, UNSW Sydney, Sydney, New South Wales, Australia; 5 Department of Infectious Diseases, Alfred Health, Monash University, Melbourne, Victoria, Australia; 6 Burnet Institute, Melbourne, Victoria, Australia; 7 Peter Doherty Institute for Infection and Immunity, University of Melbourne, Melbourne, Victoria, Australia; 8 Australian Federation of AIDS Organisations, Newtown, New South Wales, Australia; Asociacion Civil Impacta Salud y Educacion, PERU

## Abstract

We estimated the size of the population of gay-identified men who have sex with men (gay men) eligible for PrEP in Australia under the current national PrEP guidelines. Using input indicators from the *Australian Bureau of Statistics*, the national representative survey *Second Australian Study of Health and Relationships*, *and* national HIV- behavioural surveillance, we calculated the size of the population of sexually active gay men and estimated a range for the number eligible for PrEP using different scenarios based on the guidelines. In 2015, an estimated 108,850 sexually-active 16-69-year-old gay men were classified as at risk of acquiring HIV in Australia. Of these men, 10,558 to 30,913 (9.7%-28.4%) were classified as being at high risk and therefore eligible for PrEP, most commonly due to recent receptive condomless intercourse with casual partners (6.1% to 15.5%), STI infections (5.4% to 10.6%) or the use of crystal methamphetamine (1.4% to 9.4%). The higher estimates included men who may have been at HIV risk for shorter time periods or with fewer partners. Australian PrEP guidelines recommend targeting PrEP to people at high HIV risk. Our estimation of potential PrEP users informed PrEP implementation in Australia. The choice of PrEP eligibility criteria, and interpretation of the guidelines, strongly affects the population estimates. In the future, higher numbers of gay men may become eligible for PrEP, because the estimates are largely defined by and follow trends in condomless anal intercourse. Our estimation methods can be adapted to other settings.

## Introduction

Co-formulated tenofovir disoproxil fumarate and emtricitabine, (TDF/FTC, marketed as Truvada® [1]) has been widely used for HIV treatment. It has now been approved with an HIV prevention indication as pre-exposure prophylaxis (PrEP), first in the US [2] and recently in several other countries including Australia [3], where tenofovir disoproxil maleate and tenofovir disoproxil phosphate, both co-formulated with emtricitabine, have been approved also. PrEP use is supported by evidence from at least fifteen clinical trials and three observational studies which demonstrated that daily oral TDF/FTC is safe and effective in preventing HIV infection in adherent men who have sex with men, heterosexual men and women in HIV-discordant couples or recruited as individuals, and people who inject drugs [4]. In 2015, two landmark European trials, IPERGAY and PROUD, each reported an 86% HIV-risk reduction due to the use of TDF/FTC as PrEP in men who have sex with men at high risk for HIV infection [5, 6]. Results from these two studies alleviated concerns about possible poor PrEP adherence among high HIV-risk people and brought about a turning point in PrEP implementation.

Although PrEP for high-risk populations is now recommended by national or regional guidelines in the US [7, 8], Australia [9], and a number of other countries [10–12], as well as in international WHO guidelines [13], access to PrEP remains limited. In the US, universal PrEP access has not yet been achieved; only some locations (e.g., San Francisco, New York City and Seattle) have reported significant progress [14]. While PrEP is expected to have a substantial impact on HIV epidemics worldwide [15], the uptake of PrEP remains limited, partly due to the high cost of TDF/FTC [16] in settings where cheaper generics are not yet available.

One of the determinants of PrEP cost-effectiveness is how well it is targeted to those at high HIV risk. So far, there has been no universal approach to PrEP targeting. Current WHO guidelines recommend prescribing PrEP to populations with an estimated HIV incidence of at least 3 per 100 person years [13]. However, country-specific PrEP guidelines differ in their recommendations about how to target PrEP [7, 10–12, 17]. Most define PrEP eligibility rather broadly and recommend PrEP for key high-risk groups of HIV negative people.

In Australia, guidelines issued by the Australasian Society in HIV Medicine in 2015 recommended PrEP for gay men at high risk of HIV. The high-risk criteria were defined based on evidence from the Health in Men (HIM) cohort [18], which identified the highest incidence of HIV among gay men who, in the last six months, had condomless anal intercourse with an HIV positive regular sexual partner (incidence of 5.36 per 100 person-years), receptive condomless anal intercourse with an HIV-positive- or unknown-status casual partner (2.31 per 100 person-years), a diagnosis of rectal gonorrhoea or chlamydia (7.01 and 3.57 per 100 person-years, respectively), or used methamphetamine (1.89 per 100 person-years) (Table 1). Specific, easily operationalised criteria assist prescribers in conducting risk assessment and identifying individuals eligible for PrEP.

**Table 1:** PrEP behavioural eligibility criteria for high-risk men who have sex with men and their measurement.

#	Australasian society for HIV Medicine PrEP behavioural criteria, high-risk men who have sex with men (*in the last 3 months*) [9]	Self-reported indicators in 2015 GCPS which were selected to measure the criteria	
		Lowest plausible scenario	Highest plausible scenario
HR-I	Non-HIV positive man, having at least one episode of condomless anal intercourse (CLAI) with a regular HIV+ partner (not on treatment and/or detectable viral load)	In the last 6 months, having any condomless anal intercourse (CLAI)	In the last 6 months, having any condomless anal intercourse (CLAI)
HR-II	At least one episode of receptive condomless anal intercourse with any casual HIV-positive male partner or a male partner of unknown HIV status	In the last 6 months, having frequent receptive CLAI with any casual partners ^a^ AND In the last 6 months, having more than 10 sex partners	In the last 6 months, having any receptive CLAI with any casual partners ^a^
HR-III	Rectal gonorrhoea, rectal chlamydia diagnosis, or infectious syphilis	In the last 12 months, being diagnosed with a sexually transmitted infection (STI) and having an anal swab for STI or a blood test for syphilis AND In the last 6 months, having more than 10 sex partners	In the last 12 months, being diagnosed with a sexually transmitted infection and having an anal swab for STI or a blood test for syphilis
HR-IV	Methamphetamine use	In the last 6 months, using crystal meth (any account of crystal use) AND In the last 6 months, having more than 10 sex partners	In the last 6 months, using crystal meth at least monthly

Note: high risk for HIV was defined as “reporting any of the high risk factors listed below”.

**a**—HIV serostatus of casual partners was not measured in the 2015 GCPS.

Scale-up of PrEP access commenced in Australia in 2016 [19] through a series of clinical studies designed to provide interim PrEP access, while the public funding of for PrEP through the Australian Pharmaceutical Benefits Scheme (PBS) is being considered by the Pharmaceutical Benefits Advisory Committee [20]. Without the PBS subsidy, consumers have to pay the full cost of medicines.

Submissions to have medicines made available through the PBS at subsidised cost require sponsors to demonstrate that the proposed listing is cost-effective. An essential part of this assessment is to have reliable estimates of the size of the population likely to use the medication. This paper presents the estimate of potential PrEP users to inform consideration of submissions for PBS listing of PrEP and also to inform planning of PrEP access studies in various Australian States/Territories referred to earlier.

## Materials and methods

### Data sources and measures

Input indicators were obtained from the following data sources:

1. Australian Bureau of Statistics (ABS, time series spreadsheet 3101059 [21]) which provided the estimated resident male population in Australia by age in mid-2015;
2. the nationally representative Second Australian Study of Health and Relationships (ASHR2, 2013 [22]) was a source of data on sexual identity and sexual behaviour in the last 12 months;
3. the 2016 Annual surveillance report of HIV, viral hepatitis and sexually transmissible infections in Australia [23] which provided the estimated number of HIV positive men living in Australia by 31 December 2015 who acquired HIV via male-to-male sexual contact, and
4. the Gay Community Periodic Surveys (GCPS) [24, 25] conducted in 2015, which, as part of the ongoing Australian national behavioural surveillance, collect information about relationships, sexual partners and practices, and other behaviour among gay men indicative of high risk for HIV infection, such as methamphetamine use and STI diagnoses [24].

### Analyses

We estimated the size of the population of gay men at high risk of HIV infection and thereby eligible for PrEP in 2015, ahead of the start of expanded PrEP roll-out programs in Australia [26]. Calculations focused on men aged 16 to 69, as in the ASHR2 study [27]. Estimations were conducted as follows:

#### Step I: Estimation of population of sexually active gay men aged 16 to 69

The estimated resident population distribution by age and sex from the ABS was used to obtain the number of men aged 16 to 69 years in Australia in June 2015 (n_1_). From the ASHR2 survey, we obtained the proportion of Australian men aged 16–69 years old who identified as gay (1.88%) and had at least one male sex partner in the 12 months before the survey (81.9%), which was used to calculate the size of the population group of sexually active 16–69 year old gay men in Australia (n_2_ = n_1_*1.88%*81.9%). After deducting an estimated 19,097 men living with HIV who had reported male-to-male sex as an exposure category as not eligible for PrEP [23], we derived our population group of sexually active 16–69 year old gay men at risk for HIV infection (n_3_).

#### Step II: Estimation of proportion of gay men who are at “high risk” of HIV

Using GCPS data, we selected participants who were 16–69 years old, not HIV-positive by self-report (i.e., HIV-negative or of unknown HIV status) and sexually active (i.e., having had at least one male sex partner in the six months before the survey). In this subsample, we identified men at high risk of acquiring HIV, defined by 2015 Australian PrEP guidelines [17]. The measurement of each high-risk criterion was approximated by the closest available GCPS indicator with respect to a specific practice or the reference period (see Table 1):

1. A self-report of having in the last six months any condomless anal intercourse (CLAI) with an HIV positive regular male partner whose viral load was detectable was used to identify men eligible for PrEP under High-risk Criterion I (HR-I).
2. A report of receptive CLAI with any casual partners in the last six months (HIV serostatus of casual partners was not assessed by 2015 GCPS) was used to classify men as eligible for PrEP under High-risk Criterion II (HR-II).
3. A disclosure of a diagnosis of any sexually transmitted infection (STI) *and* an anal swab for STI or a blood test for syphilis in the last 12 months (direct report of anal gonorrhoea and anal chlamydia was not collected) was used to detect those eligible for PrEP under High-risk Criterion III (HR-III).
4. A self-report of crystal methamphetamine use in the last six months was used as an indicator of eligibility for PrEP under High-risk Criterion IV (HR-IV).

Because some gay men can engage in multiple risky practices, they were considered to be at high risk of acquiring HIV if they had disclosed at least one of the four factors above.

We used two scenarios to estimate a plausible range of percent of gay men eligible for PrEP.

For the lowest plausible (conservative) estimate of gay men eligible for PrEP, to meet the “high and ongoing risk” guideline requirement, we required men to: 1) have more than 10 sex partners in the last six months (except for men whose risk was from a regular partner); 2) have receptive CLAI frequently in the last six months. and/or 3) use crystal methamphetamine at least monthly in the last 6 months.

For the highest plausible (relaxed) estimate of gay men eligible for PrEP, we removed these three requirements as to the number of partners and frequency of CLAI and methamphetamine use.

#### Step III: Estimation of population of gay men who are at “high risk” of HIV

We assumed that the selected GCPS participants were representative of all adult sexually active gay men in Australia and applied the proportions calculated in Step II to the number of Australian gay men at risk for HIV derived in Step I, to estimate the number of gay men eligible for PrEP based on each of the scenarios. Uncertainty was accounted for by computing the 95% confidence intervals (95%CI) for proportions for each of the upper and lower estimates.

All analyses were performed using STATA 14.0 (StataCorp, College Station, TX, USA).

## Results

### Australian gay men at risk for HIV infection

According to ABS [21], there were n_1_ = 8,292,223 men aged 16–69 years living in Australia in mid-2015 (34.9% of the Australian population). Among participants of the ASHR2 survey, 1.88% of men aged 16–69 identified as gay and 81.9% of these reported having at least one male sex partner in the 12 months before the survey [22], so we estimated n2 = 127,947 of sexually active 16–69 year-old gay men in the population (n_2_ = n_1_*1.88%*81.9%). After excluding 19,097 gay men estimated to be living with HIV by the end of 2015 [23], the estimated number of sexually active 16-69-year-old gay men at risk for HIV infection was n_3_ = 108,850.

### GCPS participants who satisfied behavioural eligibility criteria for PrEP

In 2015, 6,676 participants of the GCPS were 16–69 year old, sexually active gay men of HIV negative or unknown status. Table 2 presents the proportions of men who satisfied the behavioural eligibility criteria for PrEP according to the lowest and highest plausible scenarios described above. Notably, only 0.02% of gay men in this sample were classified as eligible for PrEP based on having CLAI with an HIV positive regular male partner who had a detectable HIV viral load. The most common risk factor in both conservative and highest plausible estimation scenarios was receptive CLAI with any casual male partner in the last six months (6.1% (95% Confidence Interval (95%CI):5.6%-6.7%) and 15.5% (95%CI: 14.6%-16.4%) of GCPS participants, respectively).

**Table 2:** Input indicators.

Indicator	Data source	Measurement unit	Value
**Total Australian population**	ABS^b^	N	23,781,169
**Number of Australian men aged 16 to 69 years old**	ABS	N	8,292,223
**Proportion of men aged 16 to 69 years old (of the total Australian population)**	ABS	%	34.9
**Proportion of men identifying as gay among men aged 16 to 69 years old**	ASHR2^c^	%	1.88
**Proportion of gay men who were sexually active in the last 12 months**	ASHR2	%	81.9
**Number of men living with HIV in Australia who reported acquiring HIV via male-to-male sexual exposure**	ASR^d^	N	19,097
**Participants in the 2016 GCPS**	GCPS^e^	N	7,865
**Subsample of the 2016 GCPS participants who were 16–69 years old sexually active gay men of HIV negative or unknown status** ^f^	GCPS	N	6,676
**Proportion of non-HIV-positive, 16–69 year-old, sexually active gay men in GCPS, who reported behaviour consistent with:**			
***Conservative scenario*:**			
HR-I: Condomless anal intercourse with an HIV positive regular male partner (viral load detectable)		% (95% CI)	0.02 (0.01–0.03)
HR-II: At least one episode of receptive CLAI with any casual male partner in the last 6 months	GCPS	% (95% CI)	6.1 (5.6–6.7)
HR-III: STI diagnosis or positive STI test in the last 12 months	GCPS	% (95% CI)	4.9 (4.4–5.4)
HR-IV: crystal meth use, any (at least monthly) in the last 6 months	GCPS	% (95% CI)	1.4 (1.1–1.7)
more than 10 sex partners in the last 6 months	GCPS	% (95% CI)	20.7 (19.7–21.7)
Overall proportion at high risk for HIV	GCPS	% (95% CI)	9.7 (9.0–10.4)
***Relaxed scenario*:**			
HR-I: Condomless anal intercourse with an HIV positive regular male partner (viral load detectable)		% (95% CI)	0.02 (0.01–0.03)
HR-II: At least one episode of receptive CLAI with any casual male partner in the last 6 months	GCPS	% (95% CI)	15.5 (14.6–16.4)
HR-III: STI diagnosis or positive STI testing the last 12 months	GCPS	% (95% CI)	10.6 (9.9–11.3)
HR-IV: crystal meth use, any (at least monthly) in the last 6 months	GCPS	% (95% CI)	9.4 (8.7–10.1)
Overall proportion at high risk for HIV	GCPS	% (95% CI)	28.4 (27.4–29.5)

b—ABS—Australian Bureau of Statistics.

c—ASHR2—Second Australian study of Health and relationships.

d—Annual Surveillance Report 2016.

e—GCPS—Gay Community Periodic Surveys in Australia.

f—In 2016, Gay Community Periodic Surveys were conducted in New South Wales, Victoria, Queensland, South Australia, Western Australia and Tasmania.

There was a substantial overlap of risk practices. In particular, gay men who reported crystal methamphetamine use, or a recent STI diagnosis were very likely to also report receptive CLAI, and therefore adding crystal use and STIs, as criteria did not substantially increase the proportion of men eligible for PrEP. Thus, a total of 9.7% (95%CI: 9.0%-10.4%) of the selected subsample of gay men in GCPS were classified as at high risk for HIV and eligible for PrEP under the conservative scenario and 28.4% (95%CI: 27.4%-29.5%) under the relaxed scenario.

### The estimated number of gay men eligible for PrEP in Australia

The calculated number of gay men eligible for PrEP in Australia is presented in Table 3.

**Table 3:** Gay-identifying men eligible for PrEP in Australia, 2015.

	Assumed population proportion		Estimated number of gay men eligible for PrEP in Australia, per specified criteria	
	Conservative scenario	Relaxed scenario	Conservative scenario	Relaxed scenario
	% (95% CI^g^)	% (95% CI)	N_E_ (95% CI)	N_E_ (95% CI)
HR-I: Being a regular sexual partner of an HIV-infected man with whom condoms were not consistently used in the last 3 months (partner has a detectable or unknown viral load)^h^	0.02% (0.01%-0.03)	0.02% (0.01%-0.03)	22 (11–33)	22 (11–33)
HR-II: At least one episode of receptive CLAI^i^ with a casual male partner in the last 6 months ^j^^,^^k^	6.1% (5.6%-6.7%)	15.5% (14.6%-16.4%)	6,640 (6,096–7,294)	16,872 (15,892 = 17,851)
HR-III: Rectal STI diagnosis (Gonorrhoea, Chlamydia or Syphilis) during the last 12 months ^l^^,^^m^	4.9% (4.4%-5.4%)	10.6% (9.9%-11.3%)	5,334 (4,789–5,878)	11,538 (10,776–12,300)
HR-IV: Methamphetamine use in the last 3 months^n^	1.4% (1.1%-1.7%)	9.4% (8.7%-10.1%)	1,524 (1,197–1,850)	10,232 (9,470–10,994)
**Overall at high risk for HIV**^**o**^	**9.7% (9.0%-10.4%)**	**28.4% (27.4%-29.5%)**	**10,558 (9,796–11,320)**	**30,913 (29,825–32,111)**

g—95%CI– 95% Confidence Interval for proportions obtained from the Gay Community Periodic Surveys.

h—Regardless of whether the risk was ongoing.

i—CLAI–condomless anal intercourse.

j–The status of casual partners was not measured in the community samples recruited by GCPS.

k–GCPS assessed sexual practices in preceding 6 months (not in 3 months as required by PrEP eligibility criterion 2).

l–The estimate for behavioural criterion 3 is based any self-reported STI and anal swab, or blood test for Syphilis.

m–GCPS collected self-reported information about STI diagnoses in preceding 12 months (not in preceding 3 months or at screening as required by PrEP eligibility criterion 3).

n—GCPS measured use of crystal and speed. We took into account monthly or more frequent use of these drugs.

o—High risk for HIV was defined as “reporting any of the high risk factors listed below”.

Overall, we estimated that 10,558 (range: 9,796–11,320) gay men would qualify for PrEP under conservative scenario, aimed to target the highest risk men. When restrictive criteria were relaxed, the estimated number of gay men eligible for PrEP increased to 30,913 (range: 29,825–32,111).

## Discussion

We estimated that approximately 10,000 to 31,000 (9.7% to 28.4%) of gay men in Australia would be eligible for PrEP depending on the interpretation of behavioural eligibility criteria. Corrected for the uncertainty associated with the use of sample proportions, the range of gay men eligible for PrEP ranged from 9,500 to 32,000. The higher estimate included men who might only occasionally be at high-risk of HIV and men with fewer partners.

The most common behavioural eligibility criterion that was met was engaging in receptive CLAI with casual partners (6.1% to 15.5% of the estimated gay men population), and the second most common criterion was a diagnosis of an anal bacterial STI or syphilis in the last 12 months (4.9% to 10.6%). Depending on frequency of use, only 1.4% to 9.4% of gay men were eligible for PrEP based on crystal use. The prevalence of all these practices in men not on PrEP has been increasing in the past years and may increase in the future, particularly if PrEP use is associated with increasing CLAI with casual partners [25].

Based on our estimates, it appears that only a small number of men would be eligible for PrEP because they only have CLAI with an HIV-positive regular male partner who is not on treatment or has a detectable viral load. This is not surprising, as the number of HIV-positive people not on treatment and having detectable viral load is low and declining in Australia [28], and very few of them are in serodiscordant relationships.

HIV prevention programs among men who have sex with men commonly experience lack of data about the size of population for targeting—this group is difficult to quantify. Our estimates were enabled by 1) data from a nationally representative survey about sexual identity among men as well as their sexually active status [22]; 2) pragmatic PrEP guidelines and eligibility criteria which are based on evidence from local observational studies about factors that have contributed to HIV transmission among Australian gay men [17, 29], and 3) the availability of national behavioural surveillance data from gay men [24, 25].

Estimations of potential PrEP users have previously been undertaken in San Francisco [30] and nationally in the US [31]. According to the US Centers for Disease Control and Prevention about 24.7% of sexually active adult men who have sex with men in the US were estimated to be eligible for PrEP based on the 2014 US Public Health Service's PrEP clinical practice guidelines [7, 31]. This proportion falls within the range we estimated for the proportion of men who have sex with men who would be eligible for PrEP in Australia.

Our calculation of the plausible range of men eligible for PrEP will assist in estimating the cost-effectiveness of PrEP in the Australian context, and should assist in public health decision making about subsidizing PrEP.

Some local governments in Australia have already made steps towards PrEP implementation and subsidized limited PrEP access programs. The two largest PrEP access trials—Expanded PrEP Implementation in Communities in NSW (EPIC-NSW) and PrEPX in Victoria—are already actively providing PrEP to over 11,000 high-risk individuals by the end of October 2017. Both trials were informed by our estimates of PrEP eligibility at the state level.

Our analysis was limited to gay men. While certain sub-groups of heterosexual adults at high risk of acquiring HIV and people who inject drugs are also eligible for PrEP under the Australian PrEP guidelines, there are no specific indicators readily available to calculate the size of these groups. For men who have sex with men, we restricted our calculations to only those identifying as gay. Sexual practices of gay men, particularly those who engage in gay community life, have been studied in detail in Australia, because this is the population group that is most affected by HIV. Indeed, the large majority of men who have sex with men recruited by Australian studies represent gay men. Men who do not identify as gay were not included in analyses because less is known about their sexual practices and they would be substantially less likely to disclose them to a medical practitioner to obtain PrEP. We also note the limitations in our estimates associated with our input indicators from GCPS. These surveys assess practices of gay men in the preceding 6–12 months while behavioural eligibility criteria in the Australian PrEP guidelines recommend assessing practices in the last 3 months (as is more common in clinical settings). It is also important to acknowledge the uncertainty of all behavioural indicators which are self-reported and can be prone to recall and reporting biases.

The Australian approach to PrEP eligibility, and our estimation of the size of the population eligible for PrEP, was focused on individuals at high risk of HIV. The proportions eligible for PrEP (about 30% in 2015 under the relaxed scenario) appear to be close to the levels of interest and willingness to use PrEP among the Australian gay men 32]. If the cost of PrEP declines and access to TDF/FTC improves, interest in PrEP is likely to increase and PrEP targeting could be expanded to also cover people at medium-risk of HIV.

In conclusion, our relatively simple and straightforward calculation enabled evidence-based decision making about PrEP implementation in Australia and could be used to inform PrEP roll-out in settings with similar HIV transmission patterns, health services and PrEP access.
